# Implementing HIV teams to improve HIV indicator condition-guided testing in general practitioner centers in the Netherlands

**DOI:** 10.1186/s12875-024-02666-0

**Published:** 2024-12-27

**Authors:** Carlijn C. E. Jordans, Lotte Niemantsverdriet – Rokx, Jan L. Struik, Eva C. van der Waal, Paul V. J. M. van der Voorn, Nienke Bakker, Annelies Verbon, Patrick J. E. Bindels, Casper Rokx

**Affiliations:** 1 Department of Medical Microbiology and Infectious Diseases, Erasmus University Medical Center, P.O. Box 2040, Rotterdam, 3015 CN The Netherlands; 2 Gezondheidscentrum Mathenesserlaan, Rotterdam, The Netherlands; 3 Huisartsenpraktijk Händellaan, Delft, The Netherlands; 4 Huisartsenpraktijk Rozenburcht, Capelle aan den Ijssel, The Netherlands; 5 Department of Infectious Diseases, University Medical Center Utrecht, Utrecht, The Netherlands; 6 Department of General Practice, Erasmus University Medical Center, Rotterdam, The Netherlands; 7 Department of Internal Medicine, Erasmus University Medical Center, Rotterdam, The Netherlands

**Keywords:** HIV, AIDS, HIV testing, Indicator condition, General practitioner, Primary care, Family care

## Abstract

**Background:**

HIV indicator condition-guided testing is recommended by guidelines to identify undiagnosed HIV infections. However, general practitioners (GPs) frequently see patients for indicator conditions without testing them for HIV. The aim of this study was to evaluate whether implementing HIV teams, using trained GP ambassadors, promoted local HIV indicator condition-guided testing practices in urban GP centers in the Netherlands.

**Methods:**

We conducted a prospective implementation study between May 2021 and March 2023. Patients ≥ 18 years newly diagnosed with HIV indicator conditions in three GP centers were included. The intervention consisted of HIV expert led education for GPs with a stepwise implementation of point-of-care testing (phase 1), followed by adding peer-to-peer case feedback by trained GP ambassadors (phase 2). Questionnaires were used to assess the experiences and beliefs of HIV indicator condition-driven testing in patients and GPs. The primary outcome was the overall HIV testing rate in patients diagnosed with indicator conditions compared to pre-implementation. Secondary outcomes were HIV testing rate per phase and per indicator condition, HIV positivity rate, and patients’ and GPs’ experiences with this testing strategy.

**Results:**

In 132,338 patient visits, 846 (0.6%, 95%CI 0.6–0.7%) HIV indicator conditions were diagnosed, including 485 sexually transmitted infections (57.3%). Overall, 215 (25.4%) indicator conditions were tested for HIV after the implementation of HIV teams. The testing rate was comparable between the two phases (25.2% versus 25.9%, *p* = 0.83). The testing rates pre- and post-implementation were comparable (21.3% versus 25.4%, *p* = 0.33). The most frequently tested HIV indicator conditions were unexplained weight loss (*n* = 13, 41.9%), unexplained lymphadenopathy (*n* = 8, 38.1%), and sexually transmitted infections (*n* = 161, 33.2%). Three patients (1.4%, 95%CI 0.3–4.0%) tested positive for HIV. Test acceptance in patients was high as was the self-perceived knowledge of GPs on HIV indicator conditions.

**Conclusions:**

Implementing HIV teams did not enhance HIV indicator condition-guided testing in urban GP centers from a low HIV prevalence setting. The high patients acceptance rate and self-perceived knowledge among GPs regarding HIV indicator conditions did not manifest in high HIV testing rates. Patients accepted testing, but a gap was found between the self-perceived knowledge of GPs regarding HIV indicator conditions and testing, and the actual HIV testing rate.

**Trial registration:**

ClinicalTrials.gov NCT05225493 (registration date: 17-01-2022).

**Supplementary Information:**

The online version contains supplementary material available at 10.1186/s12875-024-02666-0.

## Background

Prompt identification of people with indicators for an underlying HIV infection is essential to mitigate HIV transmission and start treatment to stop disease progression. However, over half of the people newly diagnosed with HIV in Europe are late-presenters with clear cellular immunodeficiency (CD4 + T-cell count below 350 cells/mm^3^), often complicated by an acquired immunodeficiency syndrome (AIDS) [1]. A late diagnosis has significant health implications for the affected individual, with a high risk of death and reduced effectiveness of therapy. Furthermore, it facilitates HIV transmission in the population [2–6].

To end the HIV epidemic and stop transmission, the Joint United Nations Programme on HIV/AIDS (UNAIDS) has set the 95-95-95 cascade of care goals. Aiming that, by the end of 2025, 95% of all people with HIV worldwide should be diagnosed, with 95% of them accessing antiretroviral therapy (ART), and 95% of them successfully being treated with ART. The Netherlands cascade of care goals stood at 94%, 95%, and 96% respectively by the end of 2023, with an estimated 1,470 still being unaware of their HIV diagnosis [7]. Many of these people visit their healthcare providers for symptoms related to an underlying HIV infections where the infection remains unrecognized [8].

One internationally recommended key testing strategy to decrease undiagnosed HIV is indicator condition-guided testing [9–11]. HIV indicator conditions result from a weakened immune system or share a similar transmission route with HIV [10]. Patients diagnosed with HIV frequently have a history of multiple missed indicator conditions that should have prompted HIV testing by healthcare providers. This includes consultations at general practitioners (GPs) [6, 8]. Women, migrants, individuals above 50 years, and those who identify as heterosexual are at risk to remain untested when presenting with an HIV indicator condition [6, 12, 13]. These people are at risk for late presentation which, in the Netherlands and other European countries, is often diagnosed upon hospital admission for an HIV indicator condition. As HIV indicator condition-guided testing aims at unbiased HIV testing, regardless of sex, age, ethnicity, or sexual preferences, this strategy can help to identify these individuals unaware of their HIV infection and prevent late presentation [14–16].

A successful implementation of HIV indicator condition-guided testing in GP centers needs a physician recognizing indicator conditions and offering HIV testing and a patient willing to undergo testing. However, GPs reported relevant barriers on the levels of logistics (lack of time, cost of tests, laboratory availability) and skills (sexual counselling, HIV knowledge, recognizing indicator conditions, test result communication concerns) [17]. Healthcare providers may maintain misconceptions pertaining the attitudes of their patients assuming that the HIV test offer offends them or may be declined [18].

GPs play a significant role in diagnosing HIV infections in the Netherlands and other countries [19]. The study objective was to evaluate the impact of implementing HIV teams to overcome the current barriers leading to missed diagnostic opportunities. HIV teams used peer GP ambassadors to cover educational sessions, offered free point-of-care HIV tests available in local practices, and provided peer-to-peer feedback. The aim of this study was to increase HIV indicator condition-guided testing practices in urban GP centers in the Netherlands.

## Methods

### Design and participants

Prospective implementation study between May 12th, 2021 and March 31st, 2023 in three urban GP centers in the Rotterdam region, the Netherlands; Gezondheidscentrum Mathenesserlaan (GCML) in Rotterdam, GP center Händellaan in Delft, and GP center Rozenburcht in Capelle aan den IJssel. This region was chosen due to its relatively high undiagnosed HIV prevalence compared to other regions in the Netherlands [20]. An estimated total of 24,650 patients were in care at the three participating GP centers (9,000 GCML, 3,900 Händellaan and 11,750 Rozenburcht). The centers have 14 GPs permanently employed, supplemented with GPs working on a temporary basis.

Patients 18 years and older without a known HIV infection and diagnosed by their GP with one of the 13 selected HIV indicator conditions were eligible for inclusion. Selection of HIV indicator conditions (as defined by the EuroTEST guidance) was based on their prevalence in Dutch GP settings and included: community acquired pneumonia, herpes zoster, mononucleosis-like illness, psoriasis (severe or atypical), seborrheic dermatitis/eczema, sexually transmitted infections (STIs, this also included consultations for fear of STI/HIV or having a partner with STI/HIV), or any of the following unexplained conditions: chronic diarrhea, fever (lasting > 4 weeks), leukocytopenia (lasting > 4 weeks), lymphadenopathy, oral candidiasis, thrombocytopenia (lasting > 4 weeks), and weight loss [10, 14]. We used the guidelines of the Dutch College of GPs to operationalize the clinical definitions per HIV indicator condition (Supplementary Table 1).

### Intervention

The main intervention was the implementation of an HIV team which consisted of trained ambassador GPs, HIV experts, and data collectors. The HIV team used a strategy where, after an educational session to GPs led by an HIV expert before the start, two interventions were stepwise implemented: phase (1) free point-of-care HIV testing (May 12th, 2021 – September 30th, 2022), and phase (2) free point-of-care HIV testing plus the addition of peer-to-peer feedback (October 1st, 2022 – March 31st, 2023). This stepwise design was chosen to determine the effect of point-of-care HIV testing at GPs, which was identified as a knowledge gaps by the Dutch College of GPs. The educational session was given by an HIV expert of the HIV team and covered the aim of the project, the clinical definitions of HIV indicator conditions, and a demonstration of the use of point-of-care HIV tests to all GPs and GP assistants. Furthermore, all GPs received pocket cards with the selected HIV indicator conditions and their clinical definitions. Point-of-care HIV tests, Determine™ HIV early detect (Abbott), were made available in the consultation rooms of the GP centers to be used for patients diagnosed with HIV indicator conditions. For peer-to-peer feedback, the HIV team retrospectively reviewed patients files to identify cases where HIV testing was not done despite the presence of HIV indicator conditions. Information on missed opportunities to test for HIV was shared with the ambassador GP. The ambassador GP could then advice HIV testing on a case to case basis to their colleagues.

### Data collection

Data collectors from the HIV teams went on a weekly basis through all local electric health records to collect data on all newly diagnosed HIV indicator conditions, regardless whether these were recognized by the GP. The HIV team also collected pre-implementation data from the electronic health records of patients diagnosed with indicator conditions and HIV testing practice to compare HIV testing rates pre- and post-implementation of HIV teams. Pre-implementation data were collected over a 12 week period in 2019 (pre-implementation of HIV teams and COVID-19 emergence) anticipating 1,250 GP consultations weekly at a 1% HIV indicator condition yield expected to support finding meaningful differences. Data collectors discussed cases where the presence of an HIV indicator condition was uncertain with an HIV expert. Data were collected on age, sex, date of contact, diagnosed HIV indicator condition, GP, GP center, HIV test offered, HIV test performed, date of HIV test, HIV test result and reason not to test for HIV (if registered). Furthermore, GPs who recognized an HIV indicator condition entered the patient data in an electronic record (Castor) and asked these patients to complete a questionnaire. This questionnaire covered questions on experiences with this testing strategy, factors of influence for accepting the HIV test, and the testing offer by their GP. Post-implementation of HIV teams, a second questionnaire was offered to all GPs in the participating center to evaluate experiences and beliefs with HIV indicator condition-guided testing. Both questionnaires were based on the questionnaires from the evaluation toolkit of Centers for Disease Control and Prevention. (Appendix A and B) [21].

### Outcomes

The primary outcome was the overall HIV testing rate of newly diagnosed HIV indicator conditions after the implementation of HIV teams compared to the test rate pre-implementation. Patients who tested negative for HIV in the year prior to the diagnosed HIV indicator condition were considered adequately tested for HIV given that an HIV infection with such rapid disease progression (< 1 year) is highly unusual [22]. Patients with mononucleosis-like illness or STIs were only considered adequately tested if they were tested for HIV at disease presentation due to the likelihood of a recently acquired HIV infection. Secondary outcomes were the HIV testing rate per implementation phase and per HIV indicator condition. We assessed testing rates stratified by sex and age, and evaluated reasons for GPs not to test for HIV. We also assessed the HIV positivity rate and experiences and beliefs of patients and GPs with the HIV testing strategy.

### Statistical analysis

Data were described as number (%) for categorical variables and median and interquartile range (IQR) for continuous variables. HIV testing rates were assessed using the proportion of diagnosed HIV indicator conditions tested for HIV from the total of diagnosed HIV indicator conditions, including 95% confidence interval (CI). Proportions were compared using Chi-Square tests. The HIV positivity rate was assessed using the proportion of HIV indicator conditions that tested positive for HIV over the overall number of tested HIV indicator conditions. Likert scale options for the questionnaire for patients’ perspectives on HIV testing strategy ranged from ‘not at all’ to ‘a great deal’. We dichotomized outcomes for this Likert scale in two categories ‘not at all’ and ‘at least a little bit’ of influence on accepting HIV testing. For personal perspectives of participating GPs this ranged from ‘strongly disagree’ to ‘strongly agree’ and ‘never’ to ‘almost always or always’. Data was dichotomized as ‘agree’ and ‘disagree’. Missing data was collected but not included in the analysis.

A p-value ≤ 0.05 was considered significant. Data from the electronic health record and the study record forms (Castor EDC) were tabulated and aggregated in a Microsoft Excel spreadsheets (Microsoft, Redmond, WA, USA). Statistical analyses were conducted using SPSS version 28 (IBM, Armonk, NY, USA).

## Results

**Fig. 1:**
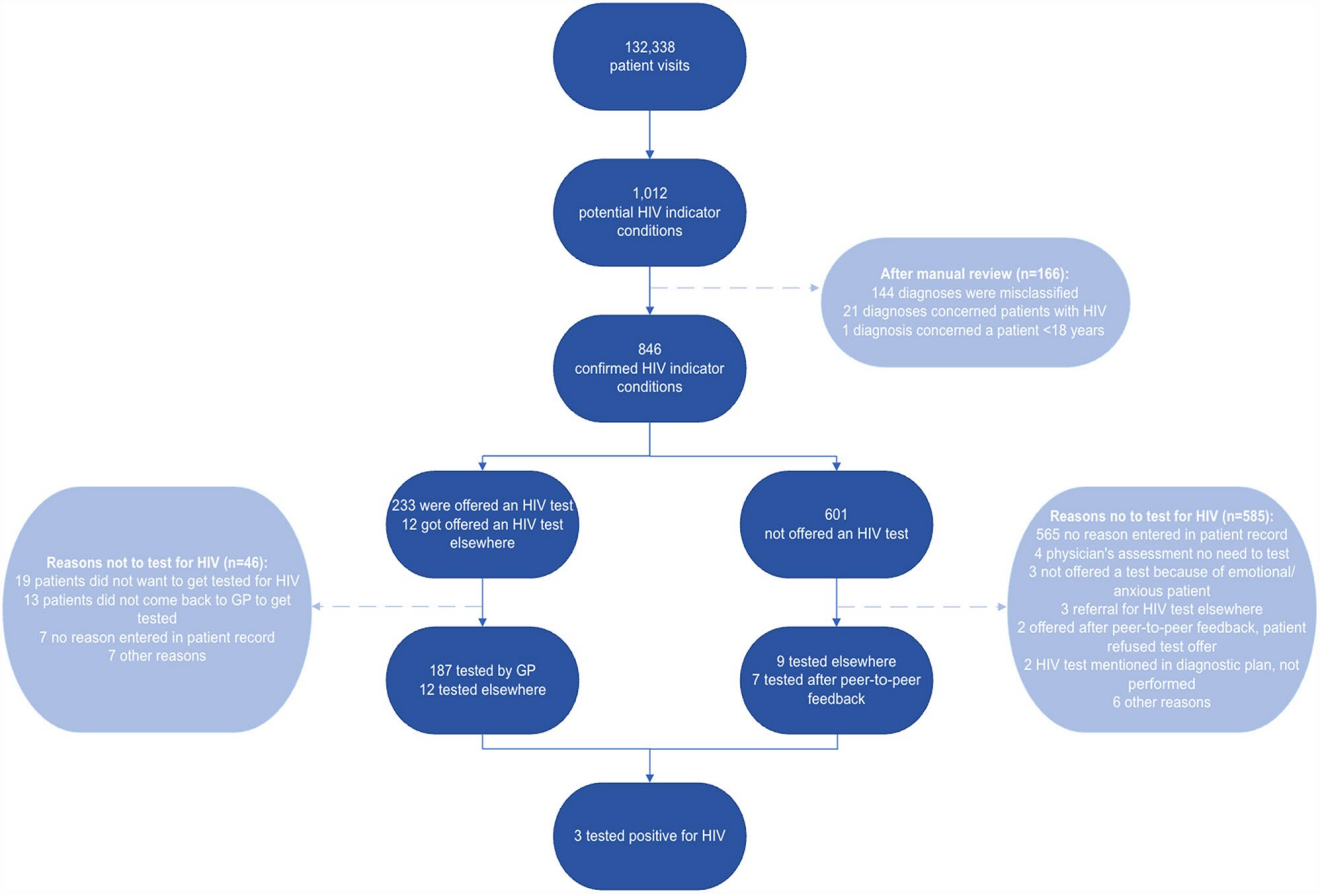
Flowchart of confirmed HIV indicator conditions, HIV testing rate, and reasons not to test for HIV. GP = general practitioner, HIV = human immunodeficiency virus

During the study period, a total of 132,338 patient visits were registered, including 52,065 visits at GCML, 24,383 visits at Händellaan, and 55,890 visits at Rozenburcht. Of these, 1,012 (0.8%) visits were registered as visits for potential HIV indicator conditions. After review, 166 (16.4%) diagnoses were excluded, foremost for misclassification as an indicator condition (Fig. 1). The remaining 846 (0.6% 95%CI 0.6–0.7%) confirmed HIV indicator conditions concerned patients (51.2% male) with a median age of 34 years (IQR: 25–55). The 17,149 visits in the pre-implementation period had a similar rate of confirmed indicator conditions (*n* = 108, 0.6%, 95% CI 0.5–0.8%) in a comparable populations (43.5% male, median age 32 years, IQR 24–52).

### Prevalence of HIV indicator conditions

Overall, the most prevalent HIV indicator condition were STIs (*n* = 485, 57.3%), herpes zoster (*n* = 122, 14.4%), and community acquired pneumonia (*n* = 101, 12.0%) in both the pre- and post-implementation period (Table 1). Unexplained fever lasting longer than 4 week was not a condition found in this study. The most frequent diagnosed STIs were chlamydia trachomatis (*n* = 124, 25.6%), condylomata acuminata (*n* = 96, 19.8%), and genital herpes simplex virus infection (*n* = 82, 16.9%). Gonorrhea and syphilis were less common, 5.2% (*n* = 25) and 2.9% (*n* = 14) respectively (Supplementary Table 2). One in twenty patients (5.1%) were diagnosed with more than one STI simultaneously.

**Table 1:** Prevalence and HIV testing rates per HIV indicator condition before and after implementation of HIV teams

	Prevalence of HIV indicator conditions		HIV testing rate	
HIV indicator condition	Pre-implementation of HIV teams, *n* (%)	Post- implementation of HIV teams, *n* (%)	Pre-implementation of HIV teams, *n* (%)	Post-implementation of HIV teams, *n* (%)
Sexually transmitted infection	69 (63.9)	485 (57.3)	20 (29.0)	161 (33.2)
Herpes zoster	16 (14.8)	122 (14.4)	0 (0)	13 (10.7)
Community acquired pneumonia	8 (7.4)	101 (12.0)	1 (12.5)	2 (2.0)
Seborrheic dermatitis/eczema	1 (0.9)	35 (4.1)	0 (0)	9 (25.7)
Unexplained weight loss	-	31 (3.7)	-	13 (41.9)
Unexplained oral candidiasis	5 (4.7)	21 (2.5)	1 (20.0)	4 (19.0)
Unexplained lymphadenopathy	1 (0.9)	21 (2.5)	0 (0)	8 (38.1)
Unexplained leukocytopenia	2 (1.9)	7 (0.8)	1 (50.0)	2 (28.6)
Unexplained chronic diarrhea	-	7 (0.8)	-	0 (0)
Severe or atypical psoriasis	1 (0.9)	6 (0.7)	0 (0)	2 (33.3)
Mononucleosis-like illness	4 (3.7)	6 (0.7)	0 (0)	1 (16.7)
Unexplained thrombocytopenia	1 (0.9)	4 (0.5)	0 (0)	0 (0)
Total	108	846	23 (21.3)*	215 (25.4)*

HIV = human immunodeficiency virus

* percentage of total HIV indicator conditions

### HIV testing rate

After implementation of HIV teams, the overall HIV testing rate of HIV indicator conditions did not significantly increase (25.4% versus 21.3% pre-implementation, *p* = 0.33). The overall HIV test offer rate by GPs was 27.5% (*n* = 233) and HIV testing was accepted by 80.3% (*n* = 187) of the patients.21 patients were tested elsewhere and 7 patients were tested after peer-to-peer feedback. Most common reasons for not testing were patient refusal or no show during medical follow-up. The testing rate was comparable between implementation phase 1 and phase 2 when peer-to-peer feedback (*n* = 209) was implemented (25.2% versus 25.9%, *p* = 0.83). The testing rates varied per HIV indicator condition. The HIV indicator conditions with the highest testing rates were unexplained weight loss (*n* = 13, 41.9%), unexplained lymphadenopathy (*n* = 8, 38.1%), and STIs (*n* = 161, 33.2%) (Table 1). The most frequently tested STIs were syphilis (*n* = 8, 57.1%), trichomonas (*n* = 5, 50.0%), and chlamydia trachomatis (*n* = 48, 38.7%). The HIV testing rate of STIs was comparable with the pre-implementation period (29.0%, *p* = 0.49). Furthermore, we observed considerable variations in overall HIV testing rate during the study period per GP center (range of 16.7–30.7%) and per GP (range from 3.5 to 49.1%) (Supplementary Table 3). Moreover, patients presenting with fear for an STI were more likely to get tested than those with a proven STI (77.8% versus 28.6%, *p* < 0.001). Patients were also more likely to get tested for HIV when they had multiple STIs (46.7% versus 27.2%, *p* = 0.03), were younger (median 29 versus 37 years, *p* < 0.001), or male (56.0% versus 49.5%, *p* = 0.03).

### HIV positivity rate

Out of 215 HIV indicator conditions that were tested for HIV post-implementation of HIV teams, three (1.4%, 95% CI 0.3–4.0%) tested positive for HIV compared to no positive HIV tests pre-implementation. Two patients were male and one was female, age between 25 and 37 years. Diagnosed HIV indicator conditions were oral candidiasis (tested by medical specialist after referral to hospital), seborrheic eczema (missed but this individual was tested because of parallel sperm donor trajectory), and a partner who tested positive for HIV (tested by GP). Two patients had prior missed opportunities for which they consulted their GP to test for HIV. The patient with seborrheic eczema was earlier diagnosed with an STI, and both the patients with seborrheic eczema and oral candidiasis were not tested for their diagnosed HIV indicator condition by their GP. Only the patient with a partner with HIV was tested adequately by their GP.

### Patients’ and GPs’ experiences

In total 65 patients who were offered a point-of-care HIV test filled in the questionnaire. The demographics of the group who completed the questionnaire were comparable to those diagnosed with an HIV indicator condition. The experience to receive point-of-care HIV testing was considered good or higher by 84.4% and most (*n* = 59, 93.7%) had understood the reason for HIV testing (Supplementary Table 4). The main reasons to accept point-of-care testing were time efficiency compared to testing at a central laboratory (*n* = 56, 86.2%) and absent costs (*n* = 50, 79.4%). A total of 18 GPs completed the questionnaire. Enhanced awareness and accessibility were recognized as benefits of point-of-care testing (Supplementary Tables 5 and 6). While 15 GPs stated to have adequate knowledge on the selected conditions and acknowledged the general benefit of HIV testing, six GPs did not consider routine HIV testing as important to GP care. Nine GPs expressed concerns that offering an HIV test could be offensive to patients, and 11 GPs said to lack adequate resources to implement HIV indicator condition guided-testing. Language barriers and patients being accompanied by relatives were also frequently mentioned as barriers, as well as time constraints.

## Discussion

Our study showed that the implementation of HIV teams, with peer-education, free point-of-care HIV tests, and peer-to-peer feedback, did not increase HIV testing rates among GPs in an urban setting in the Netherlands. However, when offered by their GP, most patients accepted HIV testing and valued the testing strategy. The beliefs disclosed by GPs revealed a discrepancy between their perceived knowledge regarding HIV indicator conditions and indicator condition-guided testing and the practical recognition and testing procedures for these conditions, alongside the identification of barriers to such practices.

HIV testing rates were generally low. Three quarters of all HIV indicator conditions remained untested. None of the individual HIV indicator conditions had a testing rate over 50%. The testing rate found in this study is comparable with other studies on HIV indicator condition-guided testing at GP centers in Western Europe [23, 24]. Education has been used in primary care to increase testing rates with some temporary effects [25, 26]. The value of point-of-care tests combined with peer-to-peer feedback was not studied before in GPs. We found that neither point-of-care testing nor peer-to-peer feedback enhanced the HIV testing rate. GPs noted barriers to communicate a post-hoc HIV test recommendation to their patient for a medical condition that had occurred weeks earlier. Real-time peer-to-peer feedback facilitates actionable feedback [27], which could likely explain the low effect. An alternative instant personal feedback strategy included direct electronic prompts which enhanced HIV testing in primary care [24]. A less individualized peer-to-peer feedback strategy using summary reports on HIV testing rate and peer comparison also increased HIV testing rates in settings with a universal HIV testing policy. This indicates that direct personal feedback could work in certain forms [28].

The barriers in time and resources to execute HIV testing found in this study are in line with other research in primary care [17]. Additionally, our findings suggest that the attitudes of GPs towards testing practices as well as unawareness of triggers to test have contributed to the low testing rate. This contradicts with the positive attitudes of GPs towards a more proactive testing strategy with immediate point-of-care testing in patients we and others have found [17]. An electronic prompt could further support the immediate testing on site. Importantly, the HIV positivity rate found in this study was above the cost-effectiveness threshold of 0.1% [29, 30]. Even if all patients who were not tested for HIV turned out to be HIV negative, the testing rate exceeded this threshold. This underlines the usefulness of this testing strategy in low prevalence areas and refutes the argument to withhold this strategy for cost-effectiveness reasons alone.

Collectively, the data support that HIV indicator condition-guided testing in primary care is clinically relevant. Effective strategies exist to enhance testing practices in primary care and should best be implemented through a setting-adapted approach [8, 31]. HIV teams can be used for this aim. Our findings indicate that these can best use educational sessions to promote GP self-efficacy and lower the practical threshold to test by using targeted electronic prompts, immediate point-of-care testing on site, and automated reflex testing for HIV. In settings with a low and declining HIV incidence, automated and more targeted testing had added value when time and personnel constraints exist with healthcare providers who also need to cover other diseases with population impact. One efficient strategy could be to integrate such service in a nurse-led practice under GP supervision with other services for infectious diseases (e.g. STI testing, pre-exposure prophylaxis for HIV [32]) and other diseases where standard care is being transferred from secondary to primary care (e.g. diabetes). This can be an attractive option from a broader patient care and economical perspective, while it also overcomes the time barriers for HIV testing imposed on GPs as a result of substituting care.

Regarding strengths and limitations, we implemented direct peer-to-peer feedback with GPs on missed opportunities to test for HIV and evaluated the value of point-of-care testing in a dense primary care network. The generalizability to settings without a comprehensive GP coverage as in the Netherlands or to non-urbanized low-prevalence settings, or high-prevalence settings is uncertain. A type 2 error cannot be excluded but the observed 4% increase in HIV testing rate after HIV team implementation, even if reflecting the true effect of the intervention, still is insufficient to make a large impact on undiagnosed HIV in the community. Inter-observer bias could have played a role with the data collectors, but the standard operating procedure and biweekly meetings with the HIV team to discuss complex cases mitigated bias. A concurrent control group in a cluster randomized design instead of using historical control would be optimal to control for unmeasured confounders. We were unable to collect data on the test offer and acceptance rate during the pre-implementation period as we used retrospective data and this information is not routinely recorded in daily healthcare practice. As the questionnaire for GPs did not collect data on the experience with peer-to-peer feedback, we were unable to report on barriers on this specific part of the implementation. Lastly, socially desirability bias may have overestimated stated knowledge on HIV indicator conditions.

## Conclusion

Implementing HIV teams with trained GP ambassadors did not enhance HIV indicator condition-guided testing in urban GP centers in the Netherlands. While patients accepted testing and reported positive experience, a gap was found between the self-perceived knowledge of GPs regarding HIV indicator conditions and testing, and the actual HIV testing rates.

## Electronic supplementary material

Below is the link to the electronic supplementary material.

Supplementary Material 1

Supplementary Material 2

Supplementary Material 3

## Data Availability

Data is available on request from the corresponding author.

## Abbreviations

AIDS: Acquired immunodeficiency virus
ART: Antiretroviral therapy
CI: Confidence interval
GCML: Gezondheidscentrum Mathenesserlaan
GP: General practitioner
HIV: Human immunodeficiency virus
IQR: Interquartile range
SKMS: Stichting kwaliteitsgelden Medisch specialist
STI: Sexually transmitted infection
UNAIDS: Joint United Nations Programme on HIV/AIDS
WMO: Wet medisch-wetenschappelijk onderzoek met mensen
