# Supplementary material for: Implementing HIV teams to improve HIV indicator condition-guided testing in general practitioner centers in the Netherlands
Source: BMC Prim Care. 2024 Dec 27;25:440. doi: 10.1186/s12875-024-02666-0 (PMC11681718; doi:10.1186/s12875-024-02666-0)
Supplement: Supplementary file 1 — Supplementary Material 1 [file 12875_2024_2666_MOESM1_ESM.docx]

**Supplementary data - Implementing HIV teams to improve HIV indicator condition-guided testing in general practitioner centers in the Netherlands**

**Supplementary table 1:** clinical definitions of HIV indicator conditions

| **Indicator condition** | **Inclusion criteria** | **Exclusion criteria** |
| --- | --- | --- |
| **Candida, oral (unexplained)** | Clinical diagnosis, confirmation by yeast culture not necessary.  The presence of Candida in the mouth / tongue / pharynx / palate.  Diagnosed macroscopically by the eye | Oral candida explained by other reasons, like   - Immunosuppressive medication (corticosteroids like prednisone, inhaled steroids, or medication used after organ transplantation) - Chemotherapy - Poorly regulated diabetes mellitus |
| **Chronic diarrhea (unexplained)** | Unexplained diarrhea lasting >4 weeks or diarrhea cause by Cryptosporidiosis, Microsporidiosis, Isosporiasis | Diarrhea explained by other reasons, like   - Immunosuppressive medication (corticosteroids like prednisone or medication used after organ transplantation) - Viral pathogens |
| **Community acquired pneumonia** | Community acquired pneumonia or second pneumonia within 12 months  Pneumonia based on clinical judgement:   - Patient with acute coughing and signs of serious illness (tachypneic, tachycardia, hypotensic, confusion) - Patient with acute coughing and moderate illness (including unilateral auscultatory deviation or CRP >100 mg/L or infiltration on X-ray or >7 days fever) | Aspiration pneumonia  Cryptogenic organizing pneumonie  Intersitial pneumonie  Obstructive pneumonie  Viral pneumonie (e.g. influenza A/B, COVID-19)  Pneumonia explained by immunosuppressant state:   - Immunosuppressive medication (corticosteroids like prednisone or medication used after organ transplantation) - Chemotherapy |
| **Fever (unexplained)** | Unexplained fever lasting >4 weeks | Fever with a clear explanation |
| **Herpes Zoster** | Clinical diagnosis by any doctor    Characterized by a painful, unilateral rash that usually appears as a band or strip following a specific dermatome of fluid-filled blisters | Primo infection with varicella zoster  Herpes Zoster explained by immunosuppressant state:   - Immunosuppressive medication (corticosteroids like prednisone or medication used after organ transplantation) - Chemotherapy - Poorly regulated diabetes mellitus |
| **Leukocytopenia (unexplained)** | Unexplained leukocytopenia lasting >4 weeks | Leukocytopenia lasting <4 weeks  Leukocytopenia with a clear explanation |
| **Lymphadenopathy (unexplained)** | Enlarged lymph nodes ≥10 mm  Enlarged lymph nodes <10 mm lasting >4 weeks  Generalized lymphadenopathy | Lymphadenopathy with clear explanation (post-infectious, malignancy) |
| **Mononucleosis-like disease**  (could be disease presentation of acute HIV infection, therefore a negative rapid point-of-care HIV test does not rule out the diagnosis of HIV, advice perform an HIV RNA test) | Acute tonsillitis with   - Fever > 7 days, or - Prolonged course of disease and inadequate result on antibiotics, or - Symptoms of M. Pheiffer: adolescence, exsudate on both tonsils, fever, painful/swollen lymph nodes in neck area, or - Acute tonsillitis in patients with risk contact for HIV in the last 3 months | Chronic tonsillitis  Positive EBV PCR or EBV serology |
| **Psoriasis (severe or atypical)** | A new, fast progressing psoriasis  Psoriasis with erythrodermia  Psoriasis with reactive arthritis  Combination of seborrheic eczema and psoriasis (sebopsoriasis)  Impetiginized psoriasis  Different phenotypes of psoriasis |  |
| **Seborrheic dermatitis/eczema** | All diagnosed cases of seborrheic eczema, and especially in case of   - Extensive or recalcitrant seborrheic eczema - Impetiginized eczema   Dandruff only in case of extensive dandruff or with limited result on treatment | Other types or excezma  ‘Normal’ dandruff |
| **Sexually transmitted infections** | Herpes simplex genitalis  Chlamydia trachomatis  Gonnorrhea  Syphilis  Trichomoniasis  Hepatitis B or C  Scabies with crustose  Multiple or recurrent condylomata  Lymphogranyloma venereum  Fear for sexually transmitted infection  Partner with diagnosed sexually transmitted infection  OR  No confirmed sexually transmitted infection, but risk contact, including  Men who have sex with men  PrEP use (follow-up)  Prostitute visit in last 6 months  People from STI-endemic countries  People with >3 different sexual partners in last 6 months  Partner belonging one of the above mentioned groups |  |
| **Thrombocytopenia**  **(unexplained)** | Unexplained thrombocytopenia lasting >4 weeks | Thrombocytopenia lasting <4 weeks  Thrombocytopenia with a clear explanation |
| **Weight loss**  **(unexplained)** | Weight loss (involuntary) >5% of weight within 6 months | Weight loss with a clear explanation, e.g. diet, increased work outs, malignancy |

**Supplementary table 2:** prevalence of sexually transmitted infections overall and per GP center

| **Sexually transmitted infection / GP center** | **GCML,**  **n (%)** | **Rozenburcht,**  **n (%)** | **Händellaan,**  **n (%)** | **Total,**  **n (%)** |
| --- | --- | --- | --- | --- |
| **Chlamydia** | 65 (23.9) | 38 (26.2) | 21 (30.9) | 124 (25.6) |
| **Condylomata acuminata** | 58 (21.3) | 26 (17.9) | 12 (17.6) | 96 (19.8) |
| **Genital herpes** | 51 (18.8) | 20 (13.8) | 11 (16.2) | 82 (16.9) |
| **Mycoplasma genitalium** | 21 (7.7) | 24 (16.5) | 5 (7.3) | 50 (10.3) |
| **Fear of STI** | 19 (7.0) | 12 (8.3) | 5 (7.3) | 36 (7.4) |
| **>1 STI** | 14 (5.1) | 9 (6.2) | 7 (10.3) | 30 (6.2) |
| **Gonorrhea** | 18 (6.6) | 5 (3.4) | 2 (2.9) | 25 (5.2) |
| **Lues** | 12 (4.4) | 1 (0.7) | 1 (1.5) | 14 (2.9) |
| **Trichomoniasis** | 5 (1.9) | 4 (2.8) | 1 (1.5) | 10 (2.1) |
| **Start PrEP** | 5 (1.8) | 3 (2.1) | 1 (1.5) | 9 (1.8) |
| **Partner diagnosed with STI** | 2 (0.9) | 3 (2.1) | 1 (1.5) | 6 (1.2) |
| **Hepatitis B** | 2 (0.7) | 0 (0) | 1 (1.5) | 3 (0.6) |
| **Total** | **272** | **145** | **68** | **485** |

GP = general practitioner, STI = sexually transmitted infection, PrEP = pre-exposure prophylaxis

**Supplementary table 3:** HIV testing rates per GP center, pre- and post-implementation of HIV teams

|  | **Pre-implementation of HIV team** | | | | **Post-implementation of HIV teams** | | | |
| --- | --- | --- | --- | --- | --- | --- | --- | --- |
|  | **Overall,**  **n (%)** | **GCML,**  **n (%)** | **Rozenburcht,**  **n (%)** | **Händellaan,**  **n (%)** | **Overall,**  **n (%)** | **GCML,**  **n (%)** | **Rozenburcht,**  **n (%)** | **Händellaan,**  **n (%)** |
| **Total** | 23 (21.3) | 16 (21.9) | 5  (14.3) | 2  (20.0) | 216 (25.5) | 130  (30.7) | 47  (16.7) | 39 (27.7) |

GCML = Gezondheidscentrum Mathenesserlaan, HIV = human immunodeficiency virus

**Supplementary table 4:** Patients’ perspectives on HIV testing strategy

| **Factor of influence on accepting HIV test today** | **Not at all,**  **n (%)** | **A little bit,**  **n (%)** | **A moderate amount,**  **n (%)** | **Quite a lot,**  **n (%)** | **A great deal,**  **n (%)** | **Missing*,**  **n (%)** |
| --- | --- | --- | --- | --- | --- | --- |
| Before the appointment, I was worried about a possible HIV infection | 41 (63.1) | 13 (20.0) | 5 (7.7) | 3 (4.6) | 3 (4.6) | - |
| I was more worried about my other health issues today | 22 (33.8) | 20 (30.8) | 13 (20.0) | 7 (10.8) | 3 (4.6) | - |
| I felt like I should take an HIV test today | 32 (49.2) | 14 (21.5) | 11 (16.9) | 4 (6.2) | 4 (6.2) | - |
| The HIV test was free of charge | 13 (20.6) | 9 (14.3) | 8 (12.7) | 7 (11.1) | 26 (41.3) | 2 (3.1) |
| The HIV test is standard of care with my health issue | 22 (34.9) | 13 (20.6) | 18 (28.6) | 3 (4.8) | 7 (11.1) | 2 (3.1) |
| I understand the reason for the HIV test | 4 (6.4) | 5 (7.9) | 16 (25.4) | 15 (23.8) | 23 (36.5) | 2 (3.1) |
| The test could be done immediately and no need to wait (long) for the result. | 9 (14.5) | 3 (4.9) | 10 (16.1) | 15 (24.2) | 25 (40.3) | 3 (4.6) |
| The test was a simple finger prick and no need to wait at the central laboratory | 9 (13.9) | 1 (1.5) | 11 (16.9) | 14 (21.5) | 30 (46.2) | - |
| I was worried someone else might learn about the result of my HIV test | 45 (70.3) | 10 (15.6) | 4 (6.3) | 2 (3.1) | 3 (4.7) | 1 (1.5) |

* missing data was calculated as percentage of total (n=65). Other data was calculated as percentage of total minus missing
GP = general practitioner, HIV = human immunodeficiency virus

**Supplementary table 5:** Personal perspectives of participating GPs about proactive HIV testing

|  | **Strongly disagree,**  **n (%)** | **Disagree, n (%)** | **Neither agree or disagree,**  **n (%)** | **Agree,**  **n (%)** | **Strongly agree,**  **n (%)** | **Missing*,**  **n (%)** |
| --- | --- | --- | --- | --- | --- | --- |
| I think routine HIV testing is an important part of regular GP care | 1 (6.2) | 2 (12.5) | 2 (12.5) | 7 (43.8) | 4 (25.0) | 2 (11.1) |
| I am concerned about the cost of HIV testing | 3 (18.7) | 10 (62.5) | - | 2 (12.5) | 1 (6.3) | 2 (11.1) |
| I am concerned that patients will be offended by being offered routine HIV testing | 1 (6.3) | 3 (18.7) | 3 (18.7) | 5 (31.3) | 4 (25.0) | 2 (11.1) |
| I am comfortable discussing HIV testing with patients | - | 1 (6.3) | 1 (6.3) | 8 (50.0) | 6 (37.4) | 2 (11.1) |
| Language barriers prevent some patients from receiving an HIV test | - | - | 2 (12.5) | 8 (50.0) | 6 (37.5) | 2 (11.1) |
| Patients are concerned about the confidentiality of routine HIV testing | 3 (18.7) | 6 (37.5) | 1 (6.3) | 3 (18.7) | 3 (18.7) | 2 (11.1) |
| I understand if my patients declines HIV testing | - | 3 (18.7) | 4 (25.0) | 7 (43.8) | 2 (12.5) | 2 (11.1) |
| Patients do not expect to be offered an HIV test at their GP | - | 5 (31.3) | 3 (18.7) | 6 (37.5) | 2 (12.5) | 2 (11.1) |
| I am concerned that a proactive HIV testing strategy will have a negative effect on patients’ opinion about our GP center | 2 (12.5) | 10 (62.5) | 2 (12.5) | 1 (6.3) | 1 (6.3) | 2 (11.1) |
| Our GP center has the resources needed to implement a proactive HIV testing strategy | 5 (31.2) | 6 (37.5) | 4 (25.0) | - | 1 (6.3) | 2 (11.1) |
| I feel that every patient with a risk factor for HIV should get tested for HIV | - | 3 (18.7) | 3 (18.7) | 4 (25.0) | 6 (37.6) | 2 (11.1) |
| It is difficult to provide the privacy needed for routine HIV testing | 5 (31.2) | 6 (37.5) | 4 (25.0) | - | 1 (6.3) | 2 (11.1) |
| I have adequate knowledge of HIV indicator conditions relevant to GPs | - | - | 1 (6.3) | 8 (50.0) | 7 (43.7) | 2 (11.1) |
| I acknowledge the benefit of HIV testing for HIV indicator conditions | - | - | 1 (6.3) | 8 (50.0) | 7 (43.7) | 2 (11.1) |

* missing data was calculated as percentage of total (n=18). Other data was calculated as percentage of total minus missing
GP = general practitioner, HIV = human immunodeficiency virus

**Supplementary table 6 :** Personal perspectives of participating GPs about proactive HIV testing

|  | **Never, n (%)** | **Rarely,**  **n (%)** | **Some-times,**  **n (%)** | **Most of the time,**  **n (%)** | **Almost always or**  **always,**  **n (%)** | **Not applicable,**  **n (%)** | **Missing*,**  **n (%)** |
| --- | --- | --- | --- | --- | --- | --- | --- |
| HIV testing interferes with providing other healthcare services | 2 (14.3) | 4 (28.6) | 5 (35.7) | - | 2 (14.3) | 1 (7.1) | 4 (22.2) |
| Patients are given HIV test results in a confidential, appropriate manner | - | 1 (7.1) | - | 4 (28.6) | 8 (57.2) | 1 (7.1) | 4 (22.2) |
| Results of HIV testing are documented and available to healthcare providers taking care of the patient | - | - | 1 (7.1) | - | 12 (85.8) | 1 (7.1) | 4 (22.2) |
| Patients are concerned or upset by the offer of HIV testing | 1 (7.1) | 3 (21.5) | 7 (50.0) | 2 (14.3) | - | 1 (7.1) | 4 (22.2) |
| The presence of family members and visitors make it difficult to discuss HIV testing with patients | - | 2 (14.3) | 3 (21.4) | 6 (42.8) | 3 (21.5) | - | 4 (22.2) |
| Patients understand the information they receive about HIV testing | - | - | - | 13 (92.9) | 1 (7.1) | - | 4 (22.2) |
| Patients who test positive for HIV receive appropriate referrals for follow-up | - | - | - | - | 9 (64.3) | 5 (35.7) | 4 (22.2) |

* missing data was calculated as percentage of total (n=18). Other data was calculated as percentage of total minus missing
GP = general practitioner, HIV = human immunodeficiency virus
