# Supplementary material for: Implementing HIV teams to improve HIV indicator condition-guided testing in general practitioner centers in the Netherlands
Source: BMC Prim Care. 2024 Dec 27;25:440. doi: 10.1186/s12875-024-02666-0 (PMC11681718; doi:10.1186/s12875-024-02666-0)
Supplement: Supplementary file 2 — Supplementary Material 2 [file 12875_2024_2666_MOESM2_ESM.docx]

**Appendix A – questionnaire for patients**

1. To what extent have you thought about your HIV risk before your appointment with the general practitioner?

|  | | Not at all | A little | A moderate amount | Quite a lot | A great deal |
| --- | --- | --- | --- | --- | --- | --- |
| A | I was worried about a possible HIV infection before the doctor’s visit. |  |  |  |  |  |
| B | Today, I was more concerned about my other health issues than about a possible HIV infection. |  |  |  |  |  |

2. How much did the following affect your decision to get an HIV test today?

|  | | Not at all | A little | A moderate amount | Quite a lot | A great deal |
| --- | --- | --- | --- | --- | --- | --- |
| A | I felt like I should take an HIV test. |  |  |  |  |  |
| B | The HIV test was free of charge. |  |  |  |  |  |
| C | The HIV test is a standard test for the conditions I went to the general practitioner for. |  |  |  |  |  |
| D | I understand the reason for the HIV test. |  |  |  |  |  |
| E | The test could be done immediately and I did not have to wait (long) for the result or call back. |  |  |  |  |  |
| F | The test was a simple finger prick and I did not have to wait at the lab. |  |  |  |  |  |
| G | I was worried someone else might learn about my test result. |  |  |  |  |  |

3. Overall, how would you rate your experience with HIV testing today?

**□** Poor

**□** Fair

**□** Sufficient

**□** Good

**□** Very good

**□** Excellent

##

4. Do you feel you are at risk for HIV?

**□** Yes

**□** No

**□** I don’t know

##

5. Are you?

**□** Male

**□** Female

**□** Would rather not say

6. What is your year of birth?

______

7. In which country were you born?

____________________

8. Describe your sexual orientation?

**□** Heterosexual

**□** Homosexual

**□** Bisexual

**□** Other, (describe) ____________________

**□** Would rather not say
