# Supplementary material for: Implementing HIV teams to improve HIV indicator condition-guided testing in general practitioner centers in the Netherlands
Source: BMC Prim Care. 2024 Dec 27;25:440. doi: 10.1186/s12875-024-02666-0 (PMC11681718; doi:10.1186/s12875-024-02666-0)
Supplement: Supplementary file 3 — Supplementary Material 3 [file 12875_2024_2666_MOESM3_ESM.docx]

**Appendix B – questionnaire for general practitioners**

1. We don’t ask for specific consent, for example, for determining kidney function. Can you also check the box for ‘HIV screening’ without asking for consent?

- Yes, because it’s routine care
- No, you need to ask for specific consent
- I don’t know

2. What is the likelihood of finding an undiagnosed HIV infection in the following patient groups?

|  | 1 in 33 (3%) | 1 in 50  (2%) | 1 in 100  (1%) | 1 in 250 (0.4%) | 1 in 500 (0.2%) | 1 in 1000 (0.1%) | 1 in 2500 (0.04%) |
| --- | --- | --- | --- | --- | --- | --- | --- |
| Herpes Zoster |  |  |  |  |  |  |  |
| Cervical dysplasia |  |  |  |  |  |  |  |
| Unexplained fever |  |  |  |  |  |  |  |
| Community acquired pneumonia |  |  |  |  |  |  |  |
| Cervical cancer |  |  |  |  |  |  |  |
| New pregnancy |  |  |  |  |  |  |  |
| Seborrheic eczema |  |  |  |  |  |  |  |
| Unexplained thrombocytopenia >4 weeks |  |  |  |  |  |  |  |

3. How many patients are you willing to test within your own general practice in order to detect and diagnose one patient with a new HIV infection in a timely manner?

patients

4. When do you think HIV testing is cost-effective?

At a prevalence of an undiagnosed HIV infection of 1 in

5. Choose one response for each of the following items that best describes your personal perspectives about routine HIV testing in your work setting.

|  | **Strongly disagree** | **Disagree** | **Neither agree nor disagree** | **Agree** | **Strongly agree** | **Don’t know** |
| --- | --- | --- | --- | --- | --- | --- |
| think routine HIV testing is an important part of regular primary health care. |  |  |  |  |  |  |
| I am concerned about cost and reimbursement for HIV testing. |  |  |  |  |  |  |
| I am concerned about cost and reimbursement for HIV testing. |  |  |  |  |  |  |
| I am comfortable discussing routine HIV testing with patients. |  |  |  |  |  |  |
| Language barriers prevent some patients from receiving routine HIV testing. |  |  |  |  |  |  |
| Patients are concerned about the confidentiality of routine HIV testing. |  |  |  |  |  |  |
| I understand it if my patient refuses an HIV test. |  |  |  |  |  |  |
| Patients do not expect to be offered routine HIV testing at their general practitioner. |  |  |  |  |  |  |
| I am concerned that routine HIV testing will have a negative effect on patients’ opinions about our general practitioner center. |  |  |  |  |  |  |
| We have the resources needed to implement routine HIV testing. |  |  |  |  |  |  |
| I believe that all patients at risk of HIV should be proactively tested |  |  |  |  |  |  |
| It is difficult to provide the privacy needed for routine HIV testing. |  |  |  |  |  |  |
| I am sufficiently informed about the HIV indicator conditions that are relevant for the general practitioner. |  |  |  |  |  |  |
| I see the benefit of HIV testing in cases of HIV indicator conditions. |  |  |  |  |  |  |

6. Circle one response for each of the following items that best describes your personal perspectives about routine HIV testing in your work setting. Please note that the response scale has changed.

|  | **Never** | **Rarely** | **About half the time** | **Most of the time** | **Almost always or always** | **Not applicable** |
| --- | --- | --- | --- | --- | --- | --- |
| Routine HIV testing interferes with providing other health care services. |  |  |  |  |  |  |
| Patients are given HIV test results in a confidential, appropriate manner. |  |  |  |  |  |  |
| Results of routine HIV testing are documented and available to health care providers taking care of the patient. |  |  |  |  |  |  |
| Patients are concerned or upset by routine HIV testing. |  |  |  |  |  |  |
| The presence of family members and visitors makes it difficult to discuss routine HIV testing with patients. |  |  |  |  |  |  |
| Patients understand the information they receive about routine HIV testing. |  |  |  |  |  |  |
| Patients who test HIV positive receive appropriate referrals for follow up. |  |  |  |  |  |  |

7. List any benefits or positive outcomes that have resulted from the implementation of routine HIV testing in your work setting.

8. List any problems or negative outcomes that have resulted from the implementation of routine HIV testing in your work setting.

9. Share any other comments about this questionnaire or about the implementation of routine HIV testing in your work setting.
